# Brain tuberculoma, an unusual cause of stroke in a child with trisomy 21: a case report

**DOI:** 10.1186/s13256-017-1258-7

**Published:** 2017-04-18

**Authors:** Abdelmoneim E. M. Kheir, Salah A. Ibrahim, Ahlam A. Hamed, Badreldin M. Yousif, Farouk A. Hamid

**Affiliations:** 1 Department of Pediatrics and Child Health, Faculty of Medicine, University of Khartoum and Soba University Hospital, P.O. Box 102, Khartoum, Sudan; 2 Department of Pathology, Faculty of Medicine, University of Bahri, Khartoum, Sudan; 3 Department of Radiology, University of Medical Sciences and Technology, Khartoum, Sudan

**Keywords:** Tuberculosis, Tuberculoma, Trisomy 21, Sudan, Case report

## Abstract

**Background:**

Tuberculosis remains a public health problem in developing countries and is associated with lethal central nervous system complications. Intracranial tuberculomas occur in 13% of children with neurotuberculosis. Patients with trisomy 21 have an increased risk for stroke, which usually stems from cardiovascular defects.

**Case presentation:**

We report a case of a 12-year-old Sudanese boy with trisomy 21 who was presented to our hospital with focal convulsions and right-sided weakness. The results of neuroimaging and histopathological examinations were consistent with cerebral tuberculoma. The patient had a good initial response to antituberculosis drugs and steroids. To the best of our knowledge, this is the first case report of multiple brain tuberculomas described in a child with trisomy 21.

**Conclusions:**

Patients with trisomy 21 have an increased risk for stroke. Our patient had an exceptional case of stroke caused by tuberculoma. The present case emphasizes the need to consider tuberculomas in the differential diagnosis of children with neurological symptoms living in areas of high tuberculosis incidence.

## Background

Tuberculosis continues to be a public health problem in the developing world. The rate of tuberculoma among all intracranial space-occupying lesions is variable [1]. Central nervous system (CNS) tuberculosis occurs in approximately 1% of all patients with active tuberculosis. It results from the hematogenous dissemination of *Mycobacterium tuberculosis* from disease elsewhere in the body and the formation of small subpial and subependymal foci in the brain and spinal cord. In some individuals, these foci rupture and release bacteria into the subarachnoid space, causing meningitis. In others, the foci enlarge to form tuberculomas without meningitis [2]. Intracranial tuberculomas occur in 13% of children with neurotuberculosis [3]. Brain tuberculomas are generally asymptomatic [4], but their symptoms depend largely on their anatomical location [5], with seizures being the commonest presenting symptom [6]. Clinical presentations are due not to tubercle bacilli or their antigens but to pressure effects of space-occupying lesions [7].

The diagnosis is established by computed tomography (CT) or magnetic resonance imaging (MRI) with or without subsequent biopsy [8]. Medical treatment is preferable to surgery, which is reserved for diagnosis or for treatment of complications [9].

We report a case of a 12-year-old Sudanese boy with trisomy 21 who was presented to our institution with focal convulsions and right-sided weakness. The neuroimaging and histopathological examination results were consistent with cerebral tuberculoma. The patient had a good initial response to antituberculosis drugs and steroids. However, the patient was subsequently lost to follow-up.

## Case presentation

A 12-year-old Sudanese boy with a known case of trisomy 21 was brought to our institution by his parents because of a 3-month history of right-sided weakness and focal seizures not associated with loss of consciousness. The boy had a history of headache but no fever, vomiting, or weight loss. He had no history of contact with a patient with tuberculosis, and he had received all his vaccines, including bacille Calmette-Guérin vaccine. The parents reported that their child had a history of multiple abdominal and cervical swellings that had been biopsied 1 year previously, but the biopsy result was inconclusive.

Clinical examination of the boy revealed that he had features consistent with trisomy 21; all of his anthropometric measurements (height, weight, and head circumference) were below the third percentile for age and sex, which is the expected finding in this child owing to short stature being associated with trisomy 21 [10] and associated tuberculosis. All his vital signs were normal. He had variable cervical and abdominal lymph nodes, which were firm but not tender. His general system examination was normal. A CNS examination of the right upper and lower limbs showed a hemiplegic gait increased tone, brisk reflexes and upgoing toe. Otherwise, the boy had intact higher functions and cranial nerves as well as a normal fundal examination result.

**Fig. 1:**
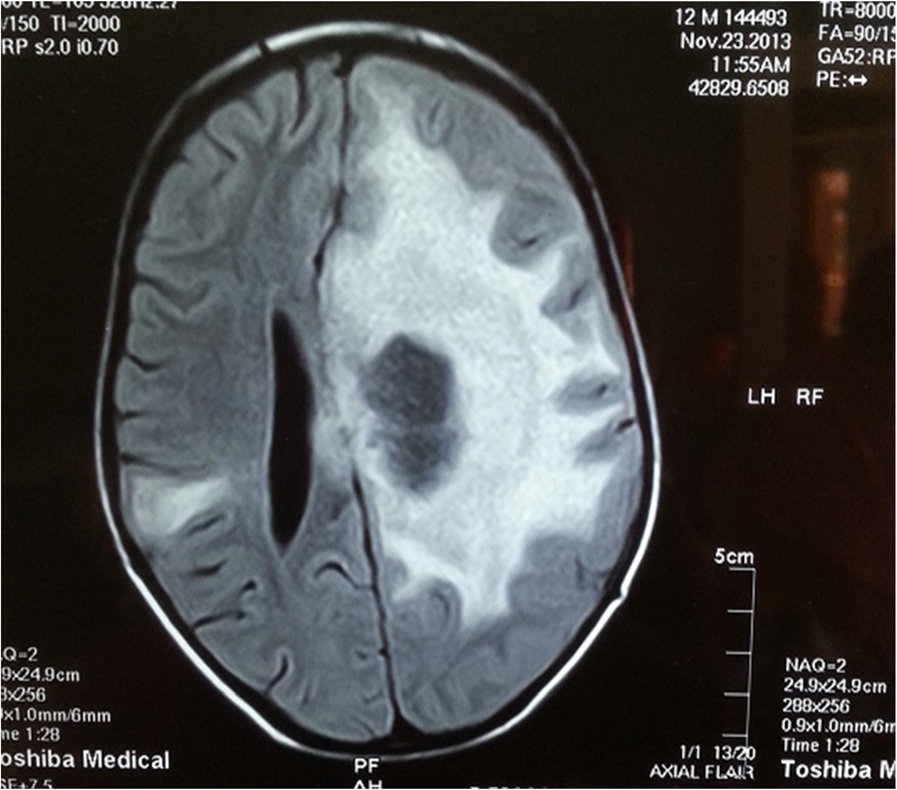
Magnetic resonance imaging of the brain showing numerous hemorrhagic mass lesions in the left temporal, left frontoparietal, and right parietal lobes

Investigations showed normal blood counts and an erythrocyte sedimentation rate of 80 mm in the first hour. The results of the patient’s renal and liver function tests were normal. His human immunodeficiency virus screening result was negative. The result of his tuberculin skin test was reactive (13 mm). His chest x-ray was normal. His echocardiogram was normal, but his abdominal ultrasound showed extensive paraaortic lymphadenopathy, which was discrete and solid with no hepatosplenomegaly. MRI of the brain showed numerous hemorrhagic mass lesions of variable size situated in the left temporal, left frontoparietal, and right parietal lobes associated with appreciable edema exhibiting vivid, amorphous enhancement after contrast dye was instilled (Fig. 1).

**Fig. 2:**
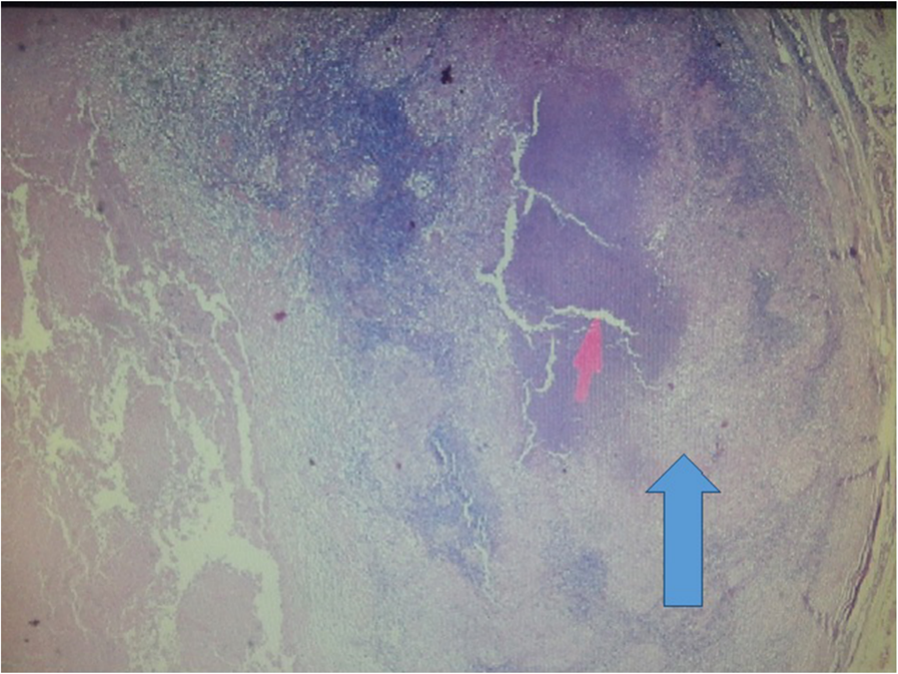
Caseous necrosis (*red arrow*) and coalescing granulomas (*blue arrow*)

**Fig. 3:**
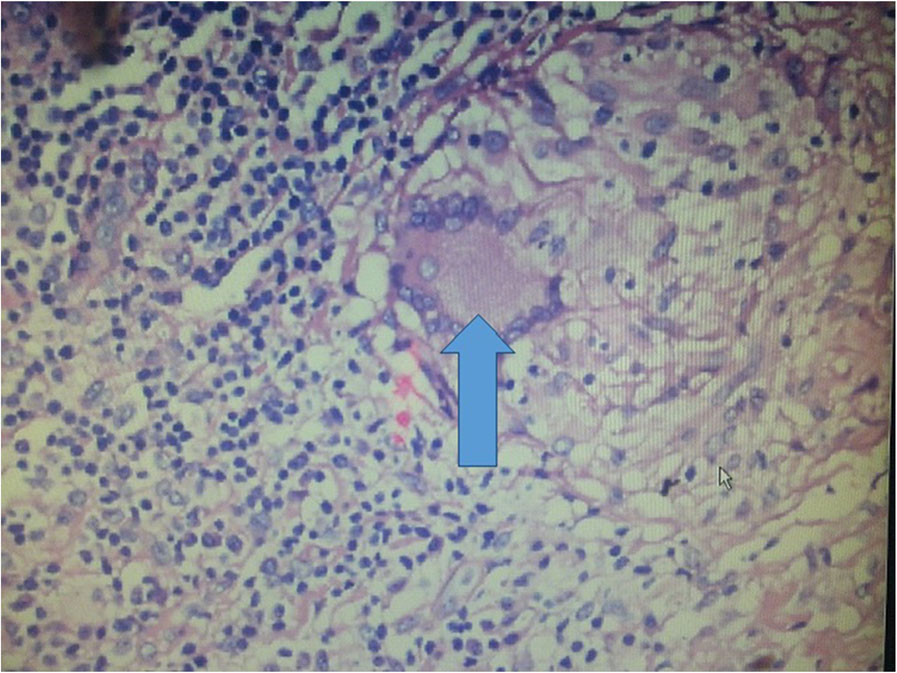
One granuloma consisting of Langerhans giant cells and adjoining aggregates of macrophages and lymphocytes (*blue arrow* is pointing to the granuloma)

Lymphoma was the suggested diagnosis, and the result of the patient’s bone marrow examination was normal. Subsequently, a cervical lymph node biopsy showed central caseous necrosis surrounded by multiple coalescing granulomas (Fig. 2) consisting of Langerhans giant cells and adjoining aggregates of epithelioid macrophages and lymphocytes (Fig. 3).

The diagnosis of brain tuberculomas was contemplated, and the child was commenced on antituberculosis treatment (isoniazid, ethambutol, pyrazinamide, and rifampicin), and the plan was to treat him for 18 months with this in addition to dexamethasone and carbamazepine. The boy showed gradual improvement in his power and gait and did not have any more seizures, and there was appreciable reduction in the size of the lymph nodes, both cervical and abdominal. The boy was discharged to home after 4 weeks. Unfortunately, the patient was subsequently lost to follow-up.

## Discussion

In developing countries, CNS tuberculosis is the most lethal complication of tuberculosis. Children under 5 years of age are at more risk of developing CNS tuberculosis [11]. The most common presenting symptom of tuberculomas are seizures [6], which is the finding in our patient, who was presented to our institution with focal seizures during the course of his illness. Moreover, in this case, neurological symptoms evolved, in the absence of fever, over a period of 3–4 months before the boy was presented to our hospital, which is consistent with the reported development of brain tuberculomas 2–6 months after infection with tuberculosis [12].

Because of the patient’s lymphadenopathy and presentation, a Mantoux test was done. The result was positive, which raised suspicion of tuberculosis. However, the diagnostic utility of skin testing for CNS tuberculosis is highly variable. Rates for children vary between 30% and 65% [12, 13], although individuals in high tuberculosis prevalence areas are more likely to have positive test results with an unrelated illness [14]. It is interesting that cerebrospinal fluid (CSF) analysis was not done for our patient, because it was refused by the parents. Moreover, routine microbiological tests for tuberculosis in the CSF have a low yield [12]. Neurocysticercosis was not considered in the differential diagnosis of our patient, owing to lack of exposure history and absence of the characteristic MRI findings showing cystic lesions demonstrating the scolex [15].

In our patient, brain MRI showed numerous hemorrhagic mass lesions in the left temporal and frontoparietal lobes. The location of the lesions is consistent with what is reported in the literature because lesions may be solitary or multiple and have a predilection to occur in the frontal and parietal lobes [16]. Brain CT was not offered for our patient, because it has a low positive predictive value [17]. CT or MRI of the brain cannot reliably distinguish tuberculoma from other causes of ring-enhancing lesions, in particular pyogenic bacterial abscess, neurocysticercosis, toxoplasmosis, or neoplasia [18].

Magnetic resonance spectroscopy is of great value in the diagnosis of tuberculoma in cases of ring-enhancing lesions seen on CT or MRI scans. It demonstrates a very high lipid peak, as well as reductions in *N*-acetylaspartate and creatinine and a choline/creatinine ratio >1.

The conventional principle in diagnosis of intracranial tuberculosis depends on a search for tuberculosis elsewhere in the body, a meticulous search for any peripheral lymphadenopathy, chest x-rays, sputum sampling for Ziehl-Neelsen staining, and ultrasound of the abdomen to look for any hepatosplenomegaly or the intraabdominal lymphadenopathy. If one or more of these are found to be positive, they can be taken as surrogate evidence favoring a diagnosis of intracranial tuberculoma [7].

To the best of our knowledge, this is the first case report of multiple brain tuberculomas described in a patient with trisomy 21. Trisomy 21 is a condition recognized to be associated with an increased risk of stroke, which in the majority of cases is secondary to cerebral embolism stemming from atrioventricular defects, right-to-left shunting, myocardial dysmotility, or valvular abnormalities. An increased proneness to meningitis and bacterial endocarditis also predisposes these patients to strokes [19]. More recently, it was suggested that strokes in trisomy 21 could also result from moyamoya disease, which is a cerebrovascular disorder characterized by progressive, noninflammatory, nonatherosclerotic occlusion of bilateral intracranial arteries [20].

Medical therapy is currently the recommended treatment for CNS tuberculosis. The regimen of isoniazid, ethambutol, pyrazinamide, rifampicin, and steroids usually results in a decrease in size and complete resolution of the tuberculoma within 3 months [21]; nevertheless, much longer treatment, up to 3 years, may be required [22]. Our patient showed gradual improvement in his power and gait, but unfortunately he and his family were subsequently lost to follow-up, which is the situation in developing counties such as Sudan, where nonadherence and loss to follow-up among patients on antituberculosis treatment could be attributed to socioeconomic and behavioral factors such as lack of transportation, cost, lack of social support, poor communication, feeling better after a few weeks of treatment, and knowledge deficit about duration of treatment [23].

Surgical resection is indicated for lesions that cause increased intracranial pressure and severe neurological deficits [24]. Bhagwati and Parulekar reported on 31 children with tuberculomas, 5 of whom needed surgical intervention; in 4 of them, the diagnosis was uncertain, and the fifth had significant mass effect in spite of treatment [25].

## Conclusions

Tuberculosis remains a public health problem with lethal CNS complications in developing countries. Patients with trisomy 21 show an increased risk for stroke, which is due to tuberculoma in exceptional cases such as our patient’s. Medical therapy with antituberculosis drugs and steroids can lead to complete resolution of tuberculomas, and surgery is reserved for selected cases. Our patient’s case emphasizes the need to consider tuberculomas in the differential diagnosis of children with neurological symptoms living in areas of high tuberculosis incidence.

## Abbreviations

CNS: Central nervous system
CSF: Cerebrospinal fluid
CT: Computed tomography
MRI: Magnetic resonance imaging
